# Surgical Management of Aortoenteric Erosion Due to Pulsatile Stress After Aneurysm Repair: A Case Report

**DOI:** 10.3400/avd.cr.20-00094

**Published:** 2020-09-25

**Authors:** Shingo Kunioka, Hiroto Kitahara, Seima Ohira, Yuki Tada, Nobuyuki Akasaka, Hiroyuki Kamiya

**Affiliations:** 1 Department of Cardiac Surgery, Asahikawa Medical University; 2 Department of Cardiovascular Surgery, Steel Memorial Muroran Hospital

**Keywords:** abdominal aortic aneurysm, small intestine, fistula

## Abstract

Secondary aortoenteric fistula or erosion (SAEFE), an abnormal connection between the aorta and gastrointestinal tract, is a rare but critical complication after abdominal aortic aneurysm repair. Most SAEFEs occur between the aorta or proximal graft anastomosis and the duodenum, and occurrence between the iliac graft and small intestine is rare. Standard SAEFE management involves graft removal and extra-anatomical bypass. However, this is extremely invasive and has a high mortality rate. We encountered a rare case of SAEFE with no sign of infection, which was successfully treated by ligating the iliac graft to reduce mechanical pulsatile stress and bleeding following the retroperitoneal approach.

## Introduction

Secondary aortoenteric fistula or erosion (SAEFE) is a life-threatening, spontaneous erosion and irregular communication between the graft and the gastrointestinal tract after surgical repair of abdominal aortic aneurysm. The reported incidence of SAEFE including graft-enteric fistula is 0.6%–1.6% after aortic reconstructions.^1,2)^ It has been reported that 90% of SAEFEs are aortoduodenal, 7.4% are aortojejunal, and 4.3% are aortoileal.^3)^ To the best of our knowledge, few papers have discussed iliac graft to ileal fistula,^3,4)^ and there is no report describing the surgical management of this condition. We encountered a rare case of SAEFE that was treated by ligating the distal limb of the graft 4 years after the aortic reconstruction.

## Case Report

**Figure 1:**
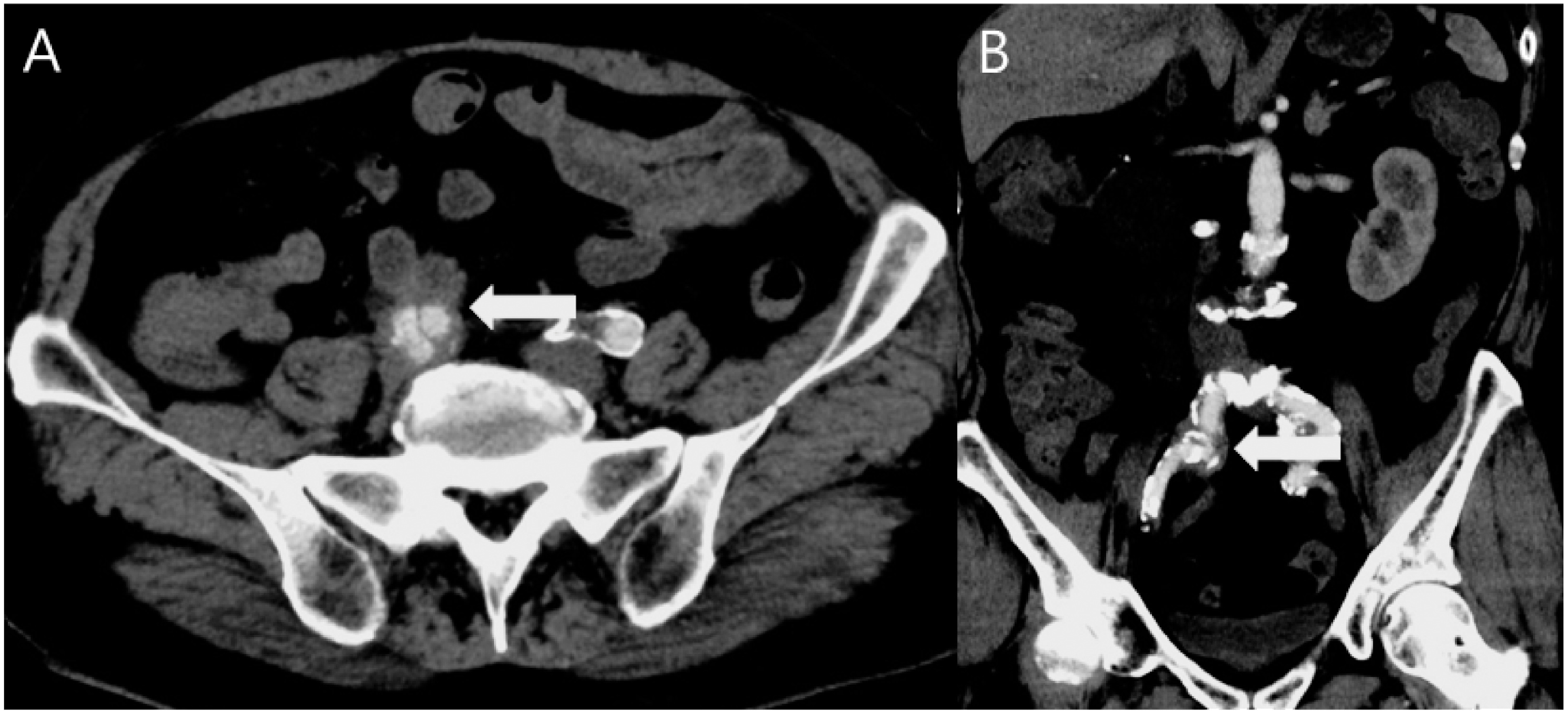
Fig. 1 Preoperative images. (**A**), (**B**) Enhanced computed tomography showed no extravasation and showed the adhesion between the iliac branch and small intestine (white arrow).

**Figure 2:**
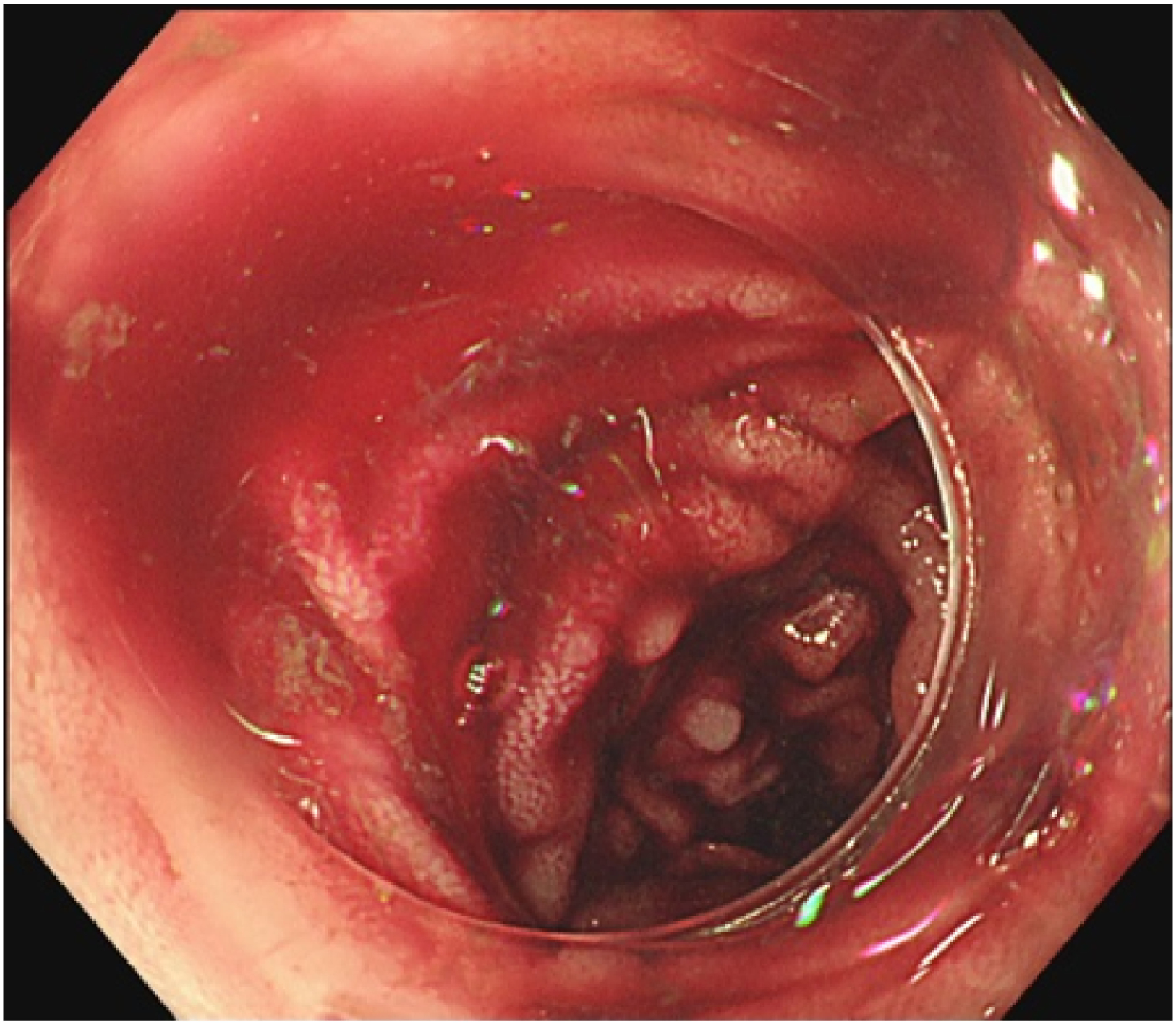
Fig. 2 Preoperative double-balloon enteroscopy findings. The view is seen from the anal site. Fresh blood was found coming from the proximal ileum.

**Figure 3:**
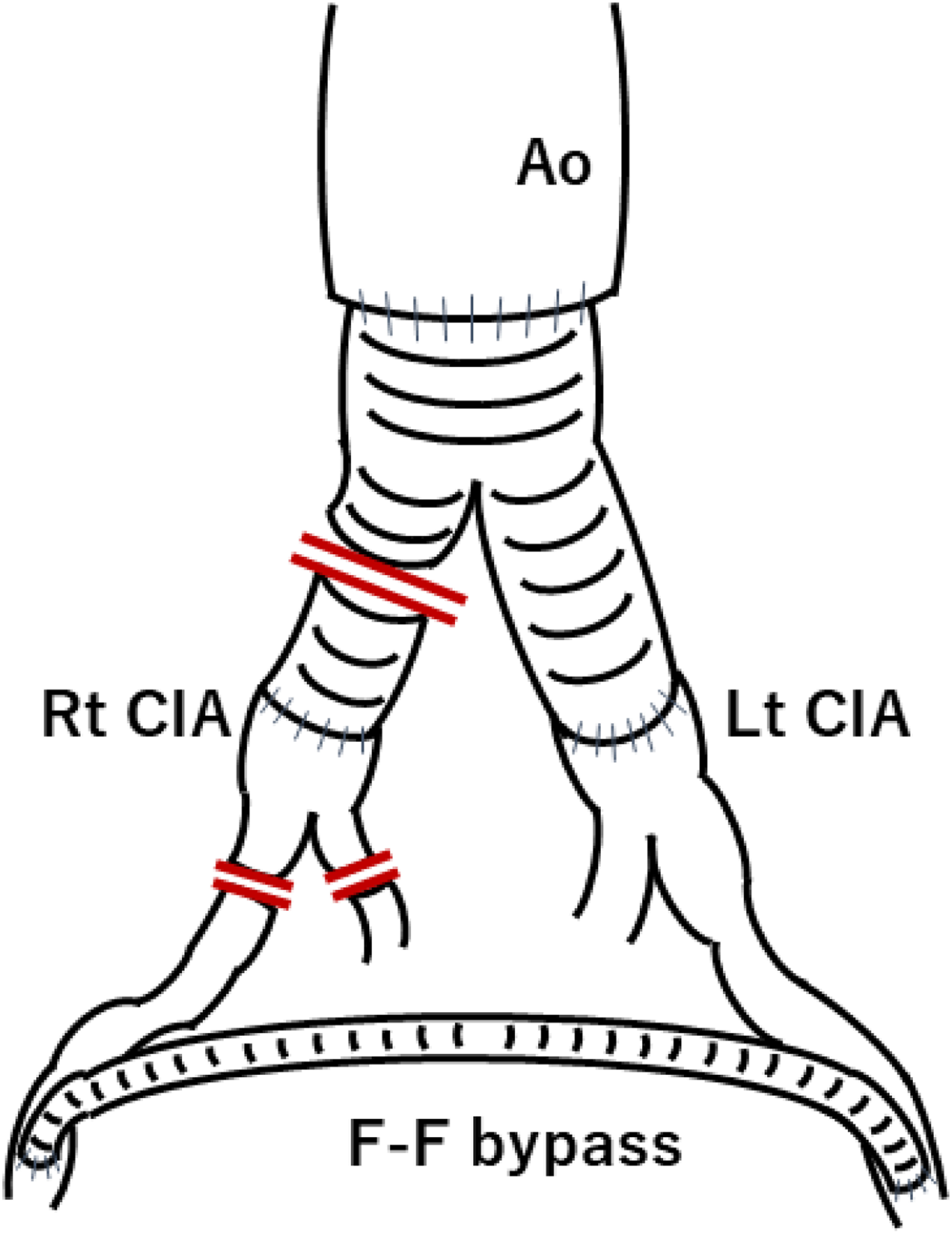
Fig. 3 The schematic diagram of the operation. The red lines show the sites of ligation of the graft and artery.

A 72-year-old man complaining of repeated gastrointestinal bleeding was transferred to our institution. He had a history of abdominal aortic aneurysm and aortic reconstruction using a vascular prosthesis at another hospital. Computed tomography (CT) imaging showed no active bleeding and adhesion between the right iliac graft and the small intestine (**Figs. 1A** and **1B**). A double-balloon enteroscopy showed fresh blood coming from 40 cm above the Bauhin’s valve, suggesting the existence of small intestinal hemorrhage (**Fig. 2**). This was at the same location as the adhesion between the small intestine and the right iliac branch of the graft, demonstrated by CT imaging. The decision was made to intervene with surgical management because of the hypothesis that the gastrointestinal bleeding was due to erosion of the small intestine, which was likely caused by mechanical pulsatile stress of the iliac branch of the graft. He did not show any signs of inflammatory reaction; his body temperature was normal; and blood examination did not show the elevation of the number of white blood cells and C-reactive proteins (6900/µl, 0.04 mg/dl). Initially, femoro-femoral artery bypass grafting was performed. The proximal part of the right iliac branch of the graft and the external and internal iliac arteries were then ligated through the retroperitoneal approach to reduce pressure in the graft (**Fig. 3**). Intraoperative findings indicated that the adhesion between the graft and the small intestine was dense, and there was no sign of infection, such as purulent drainage or abscess cavity. Therefore, resection of the small intestine was not performed, and the graft was covered with connective tissue around retroperitoneum. The postoperative course was uneventful, without any sign of recurrent bleeding or infection, and the patient was discharged 1 month after surgery. There has been no recurrence of bleeding or infection upon postoperative 1 year of careful follow-up.

Ao: abdominal aorta; CIA: common iliac artery

## Discussion

We encountered a rare but life-threatening complication, SAEFE between the distal iliac graft and small intestine, which was successfully managed by ligating the graft and eliminating the mechanical pulsatile stress without small intestine resection. The mortality of SAEFE is reported to be 50%–70%,^2,3)^ and it remains as one of the critical complications after aortic vascular surgery. The pathogenesis of this condition is not clear; several reports have indicated that this might be caused by gradual erosion of the pulsatile noncompliant aortic graft into the bowel, with or without infection through the intestinal contents.^1,5)^

The diagnosis of SAEFE is not necessarily easy as no typical symptoms have been identified. Gnus et al. described 24 patients with SAEFE; 16 of which presented with symptoms of gastrointestinal bleeding, 6 with hemorrhagic shock, 4 with septic shock, 8 with abdominal pain, 4 with back pain, and 8 with a pulsatile abnormal mass.^6)^ Some reports have suggested the existence of herald bleeding, which is a characteristic warning sign of this disease.^1,7)^ Song et al. suggested that the frequency of herald bleeding was an average of 3.6 episodes and that the time between the initial herald bleeding and massive exsanguination ranged from 5 h to 5 months (median, 4 days, with over 50% of cases being longer than 3 days).^8)^

The management of SAEFE varies and is dependent on the patient’s condition, with complete resection of the fistula being a standard treatment option. Nevertheless, the surgical mortality rates of SAEFE remains high. Several reports have argued that SAEFEs can be divided into two groups: direct communication between the graft and intestinal lumen and periprosthetic fistula due to an eversion of an intestinal loop.^2,9)^ Some reports recommend complete graft resection as treatment of SAEFE with infection and establishment of extra-anatomical circulation.^1,5)^ However, if the adhesion is dense, a broad range of adhered small intestine resections are required, and this can cause some various complications, such as the short bowel syndrome. Contrarily, Shindo et al. reported spontaneous closure of graft-duodenal fistula without any surgical fistula closure.^10)^ This report suggested that if infection was controllable during the pre- and post-operative period, then there is a possibility of spontaneous closure of the fistula. In our case, the patient did not show any sign of infection; therefore, we decided to perform simple ligation of the iliac graft to reduce pulsatile stress. The postoperative course was uneventful without recurrent hemorrhage or infection. This finding supports the hypothesis that gradual mechanical stress can cause bowel erosion. This suggests that if SAEFE is caused by erosion of the small intestine, simple ligation of the graft to reduce the pulsatile stress is effective treatment if the patient shows no signs of infection.

## Conclusion

We successfully performed surgical repair of SAEFE without any signs of infection before the occurrence of life-threatening bleeding using simple ligation of the iliac graft. If there is no sign of infection, this less invasive surgical treatment may be an effective alternative to removal of the whole graft and intestine.

## Additional Note

The patient and all authors provided informed consent for publication of the study.

## References

[1] Champion MC, Sullivan SN, Coles JC, et al. Aortoenteric fistula. Incidence, presentation recognition, and management. Ann Surg 1982; 195: 314-7. 7059240 10.1097/00000658-198203000-00011 PMC1352637

[2] Elliott JP Jr, Smith RF, Szilagyi DE. Aortoenteric and paraprosthetic-enteric fistulas: problems of diagnosis and management. Arch Surg 1974; 108: 479-90. 4815922 10.1001/archsurg.1974.01350280083014

[3] Bunt TJ. Synthetic vascular graft infections. II. Graft-enteric erosions and graft-enteric fistulas. Surgery 1983; 94: 1-9. 6857503

[4] Deijen CL, Smulders YM, Coveliers HME, et al. The importance of early diagnosis and treatment of patients with aortoenteric fistulas presenting with herald bleeds. Ann Vasc Surg 2016; 36: 28-34. 27423720 10.1016/j.avsg.2016.03.028

[5] Busuttil RW, Rees W, Baker JD, et al. Pathogenesis of aortoduodenal fistula: experimental and clinical correlates. Surgery 1979; 85: 1-13. 153003

[6] Gnus J, Ferenc S, Kościelna M, et al. Secondary aortoenteric fistula after abdominal aortic graft implementation in our own material. Adv Clin Exp Med 2016; 25: 1265-71. 28028982 10.17219/acem/66621

[7] Hashimoto M, Goto H, Akamatsu D, et al. Long-term outcomes of surgical treatment with in situ graft reconstruction for secondary aorto-enteric fistula. Ann Vasc Dis 2016; 9: 173-9. 27738458 10.3400/avd.oa.16-00082 PMC5027253

[8] Song Y, Liu Q, Shen H, et al. Diagnosis and management of primary aortoenteric fistulas-experience learned from eighteen patients. Surgery 2008; 143: 43-50. 18154932 10.1016/j.surg.2007.06.036

[9] Vollmar JF, Kogel H. Aorto-enteric fistulas as postoperative complication. J Cardiovasc Surg (Torino) 1987; 28: 479-84. 3654732

[10] Shindo S, Inoue H, Motohashi S, et al. Spontaneous evacuation of a vascular metallic stent through a graft-duodenal fistula with concomitant non-surgical fistula closure. Ann Vasc Dis 2016; 9: 338-41. 28018510 10.3400/avd.cr.16-00068 PMC5174998

